# Anti-reflecting metasurface for broadband polarization independent absorption at Ku band frequencies

**DOI:** 10.1038/s41598-022-24691-8

**Published:** 2022-11-22

**Authors:** Muhammad Amin, Aliza Fida, Aamir Rashid, Omar Siddiqui, Farooq A. Tahir

**Affiliations:** 1 College of Engineering, Taibah University, 41411 Madinah, Saudi Arabia; 2 Department of Electronics Engineering, University of Engineering and Technology, Taxila, Pakistan; 3 School of Electrical Engineering and Computer Science, National University of Sciences and Technology (NUST), Islamabad, Pakistan

**Keywords:** Energy science and technology, Engineering

## Abstract

An impedance matched metasurface can efficiently channel the electromagnetic fields for maximum power transfer. The thin film based impedance matching techniques often utilize highly dissipative materials and destructive interference of reflection components from multiple subwavelength layers. Here, we propose a novel method to achieve anti reflection characteristics through destructive interference of antiparallel electromagnetic scattering emerging from chiral metasurface. The supercell structure of metasurface consists of four adjacent multi split-rings on FR-4 substrate. The split-rings are arranged to induce anti-parallel surface currents leading to destructive interference for scattered fields. The antireflection characteristics results in near perfect broadband absorption at dual frequency bands. A broadband absorption of 983 MHz is achieved between 12.687 and 13.669 GHz. Similarly, a narrow band absorption of 108 MHz is achieved in frequency range of 15.307–15.415 GHz. The impedance matched with unique symmetric design of supercell results in identical absorption for both x- and y-polarized incident fields. The numerical and experimental results verify broadband absorption characteristics at Ku band frequencies. The proposed metasurface absorber can be used for microwave energy harvesting applications.

## Introduction

Recent advances in material science and fabrication technology enabled precise control over propagation of electromagnetic waves including its spectral and polarization properties^1,2^. Many practical applications are realized by manipulation of electromagnetic fields including sensing^3–5^, imaging^6,7^, polarization modulation^8–12^, and modulation of light^13^. Salisbury screen is one of the promising application that prohibits the scattering by impedance matching the surface with the surrounding medium^14^. Salisbury screen was one of the first attempts to reduce the radar cross sections of fighter aircraft^15^. It relies on scattering paths from multiple layers to supress the overall reflection. However, their spectral absorption is limited and absorption operates around single frequency only^16,17^. Besides impedance matching through dissipation losses contributes significantly to the performance of absorbers.

During the last two decades metamaterials of various types were proposed to support effective control over electromagnetic fields^18,19^. Several resonant metamaterial designs were proposed to minimize the radar cross section of scattering objects^20–22^. The impedance matching of the resonant metasurface is the key to support the anti-reflection characteristics^23–25^. Metasurface absorber comprising of complicated geometry build over split ring patch achieves negative refractive index that leads to near perfect absorption at discrete frequencies around X and Ku bands^26^. The logarithmic spiral resonator embedded with lossy materials are used to absorb incident microwave energy between 6 and 37 GHz frequencies^27^. Similarly, the ultrabroadband of microwave radiation were absorbed by metamaterial array of conical shaped saline water columns^28^. The absorption method relies on the intrinsic absorption coefficient of saline water and the absorbed energy is dissipated as heat^28^.

In recent years, a lot of work is theoretically and practically demonstrated in metamaterial absorbers (MMA)^29^. The first MMA was demonstrated by Landy et al in 2008 that had the capability of fully absorbing EM waves using a single layer unit cell^20^. Since then, metamaterials are widely used in the microwave, infrared, terahertz and optical frequency ranges^30^. MMA is designed by incorporating the metallic structure with the dielectric medium to attain electromagnetic resonances. Therefore, the thickness of the dielectric medium and the geometry of the top metallic plate are important for generating electromagnetic resonances. MMA is widely used in the microwave regime for various applications such as in micro-bolometers, anechoic chamber, scattering reductions^31^, thermal sensing^32^, and in energy harvesting^33^. Recently, perfect absorbers operating at infrared frequencies are designed using all dielectric semiconductor materials^34^. The design consists of semiconductor resonators positioned in the direction of wave vector meanwhile embedded in low refractive index medium. Artificial microwave blackbody structure is proposed based on similar design of resonator element placed on top of opaque metallic ground plane^35^. Such blackbody elements offer a unique way to design tunable microwave absorbers. A metasurface design based on detuned resonator elements are utilized for broadband absorber at X band frequencies^36^. The approach is based on selection of absorber elements placed closed to each other having different sizes of their unit cells. The selected absorbers are having resonant absorption frequencies close to each other such that the overall metasurface absorption is around X-band frequencies. The absorption bandwidth for 12 units and 16 units is optimized to nearly 2.73 GHz and 2.55 GHz of a minimum 80$\%$ absorption level.

The first MMA proposed by Landy et al is both polarization-sensitive and has a narrow bandwidth that restrict its application. It is desirable to achieve broadband for various applications. Broadband can be realized by a multilayer design but it increases the overall thickness of the design and makes the alignment of layers more difficult^37^. Lumped elements can also be used for this purpose but they require additional fabrication which can degrade absorber performance^38–40^. As an alternative to these designs, single layer structures have been studied and found to be more suitable. Single layer MMA reported in the literature are either narrowband, polarization-sensitive or not angular stable. However, few broadband single-layer MMA has been reported so far^37,40^.

Wireless energy transfer and energy harvesting methods are expected to become part of next generation power transmission system^41,42^. Recently, there is interest in RF energy harvesting applications by use of metamaterial absorbers^43–46^. The RF energy harvesting relies on efficient rectification of high frequency electromagnetic energy. This means that conventional dissipative materials can no longer contribute to efficient RF energy harvesting application^47^. Therefore, there is renewed interest in resonant absorption methods by impedance matching with low loss materials. Recently, resonance based chiral metasurfaces are proposed to transform polarization of electromagnetic waves. A microwave metasurface is designed to offer a simultaneous linear to cross-polarization conversion and linear to circular polarization conversion^48^. The bow-tie shaped unit cell element design supports large operational bandwidth (for polarization conversion of reflected fields) at microwave frequencies. The chiral metasurface operate through breaking structural symmetry that allows the surface currents to flow asymmetrically. The induced displacement currents lead to cross polarized components in the radiated far field^49,50^.

In this paper, we propose a novel scheme to achieve antireflection characteristics in microwave metasurface without the need of highly dissipative substrates^28^. The method to achieve antireflection characteristics is based on interference of antiparallel polarization states from adjacent scattering elements. The polarization states are controlled at subwavelength scale by utilizing periodic supercell of chiral elements constituting metasurface. The antireflection characteristics are demonstrated by near perfect absorption around Ku band frequencies.

**Figure 1:**
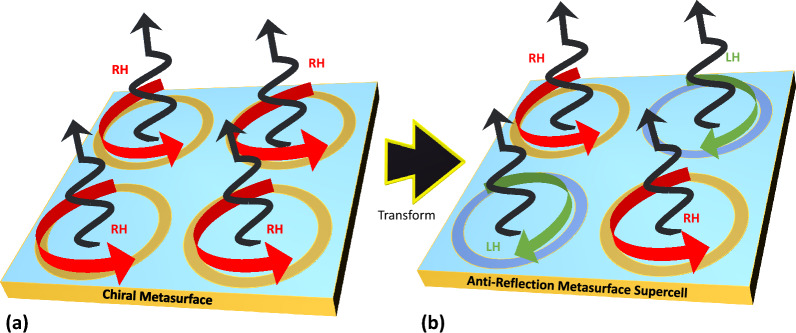
Schematic illustration of transformation of chiral reflecting metasurface cells consisting of four adjacent unitcells of anti reflecting metasurface supercell. Rotating arrows (left or right handed) on the surface represent current direction. Black arrows represent radiated fields emerging from each unit cell.

## Anti-reflection metasurface based on out-of-phase reflection from supercell

The antireflection properties that contribute to the overall absorption characteristics relies on induced currents on individual unitcells. Figure 1a shows four adjacent unit cells inside a conventional polarization conversion metasurface^48,49^. Here, each unit cell supports rotational currents in response to the incident electric fields. Such metasurfaces are used as waveplate to transform incident fields to orthogonal or circularly polarized reflected fields. If the distance (*p*) between unit cells is significantly smaller compared to wavelength of interest i.e., $p<<\lambda$, the resulting chiral reflection can be suppressed by transformation of two of the four unit cells to radiate out of phase fields compared to original unitcell as shown in Fig. 1b. Therefore, the four unit cells form two pairs due to out of phase chiral reflected fields exhibiting either LHC or RHC polarization states. Hence, the four adjacent unit cells are considered as supercell with antiparallel polarization states leading to destructive interference for scattered fields.

**Figure 2:**
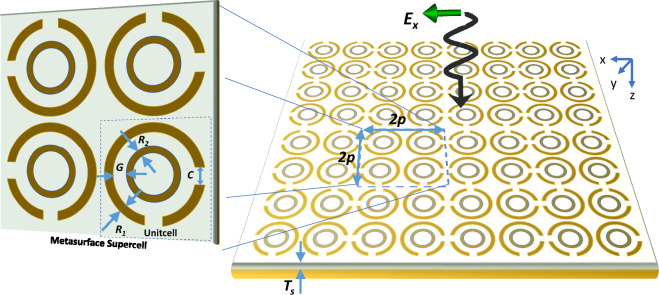
Schematic design illustration of anti reflecting metasurface on top of FR-4 substrate backed by metallic ground plane. Inset shows supercell arrangement of four adjacent elements of split rings in mirror symmetric arrangement.

## Design of anti-reflecting metasurface structure

The design of anti-reflecting metasurface relies on efficient conversion of linearly polarized incident fields to circularly polarized reflected fields. The proposed design involves mutli-ring structure with multiple splits on the outer ring as shown in Fig. 2. The outer rings having directional splits allows the generation of directional surface currents over the unit cells. A supercell consisting of four adjacent multi-split ring structures are arranged to achieve four-fold symmetry. The perturbations induced within the adjacent cells allows out of phase fields to achieve suppression of scattered waves. The split ring metasurface resides on thin dielectric substrate of thickness $T_s$ . The geomertical design parameters provided in Fig. 2 are period *p*, radius $R_1$, gap between rings *G*, and split-ring gap size *C*. The structural parameters are summarized in Table 1.

**Table 1:** Specification of geoemetric parameters described in Fig. 1.

Parameters	Dimensions (mm)
p	11
${R}_1$	0.96
${R}_2$	0.4
${T}_s$	1.6
*C*	2.05
*G*	1

The FR-4 material is used as substrate with relative permittivity of $\epsilon _r =$ 4.4 and loss tangent of 0.02. Metasurface is formed by a patterned layer of ultrathin copper sheet with electrical conductivity of $\sigma _{copper}=5.8 \times 10^7$ S/m and thickness of 35 μm. The full wave simulation of metasurface is carried out using CST Microwave Studio using periodic boundary conditions along x- and y-directions. The electromagnetic simulation uses ground plane of sufficient thickness larger than skin depth to block transmitted fields on other side of metasurface.

## Results

**Figure 3:**
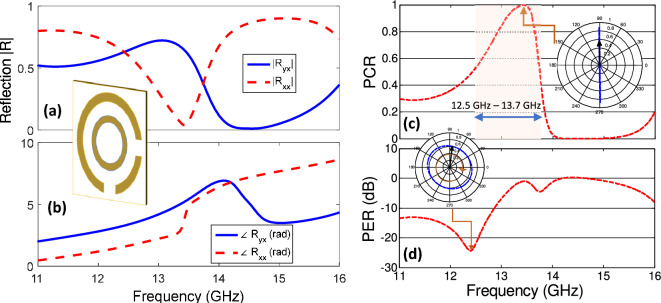
(**a**) Magnitude and (**b**) phase of reflection coefficients for co- $R_{xx}$ and cross $R_{yx}$ polarized components for co-direction chiral reflecting metasurface. (**c**) Polarization extinction ratio in dB for co-direction chiral reflecting metasurface. Inset for figure (**c**) and (**d**) shows the contour of time varying orientation of reflected electric field vector at resonance frequency for cross and circular polarization.

It will be interesting to analyze the response of perturbation free metasurface with all identical unitcells. The unidirectional cells possess structural symmetry along the diagonal axis. The response of metasurface with unidirectional unitcells is provided in Fig. 3a,b. The coefficients of Jones matrix provides useful description to analyze the effect of polarization conversion.

1 $$\begin{aligned} \begin{bmatrix} E_{xr} \\ E_{yr} \end{bmatrix} =\begin{bmatrix} R_{xx} &{} R_{xy} \\ R_{yx} &{} R_{yy} \end{bmatrix}\begin{bmatrix} E_{xi}\\ E_{yi} \end{bmatrix} \end{aligned}$$

Here, $E_{xi}$ and $E_{yi}$ are incident fields and $E_{xr}$ and $E_{yr}$ are reflected field components. Considering x-polarized incident fields the complex Jones matrix coefficients $R_{xx}$ and $R_{yx}$ shows that the transformation of incident fields to co- and cross polarized reflected fields respectively. Figure 3a,b provides the coefficients of Jones matrix components for x-polarized incident fields.

The Polarization Conversion Ratio (PCR) can be calculated using coefficients of jones matrix. The PCR provides ratio describes the ability of cross polarization conversion and maintained between 0 to 1.

2 $$\begin{aligned} \text {PCR} = |R_{yx}|^2\big /(|R_{yx}|^2+|R_{xx}|^2), \end{aligned}$$

The induced currents on the metasurface leads to cross polarized reflection that can be calculated using Eq. (2). As shown in Fig. 3c, the Polarization Conversion Ratio (PCR) maintains above $50\%$ between 12.5 and 13.7 GHz. The inset in Fig. 3c shows that x-polarized incident fields are converted to y-polarized reflected fields. Furthermore, the metasurface can also be investigated for circular polarization conversion. For this purpose we can define polarization extinction ratio (PER) using coefficients of jones matrix. The PER coefficients calculates efficiency as the ratio between right ($|R_{xx}+iR_{yx}|$) to left- ($|R_{xx}-iR_{yx}|$) handed circularly polarized fields calculated in logarithmic scale (in dB).

3 $$\begin{aligned} \text {PER} = {20 \log }_{10}(|R_{xx}+iR_{yx}|\big /|R_{xx}-iR_{yx}|)\, \end{aligned}$$

Figure 3d provides polarization extinction ratio calculated using Eq. (3). It is clear that the unidirectional unicells for the metasurface supports circularly polarized fields at 12.4 GHz frequency. This is further supported by analyzing the inset in Fig. 3d shows that x-polarized incident fields are converted to left handed circularly polarized reflected fields.

**Figure 4:**
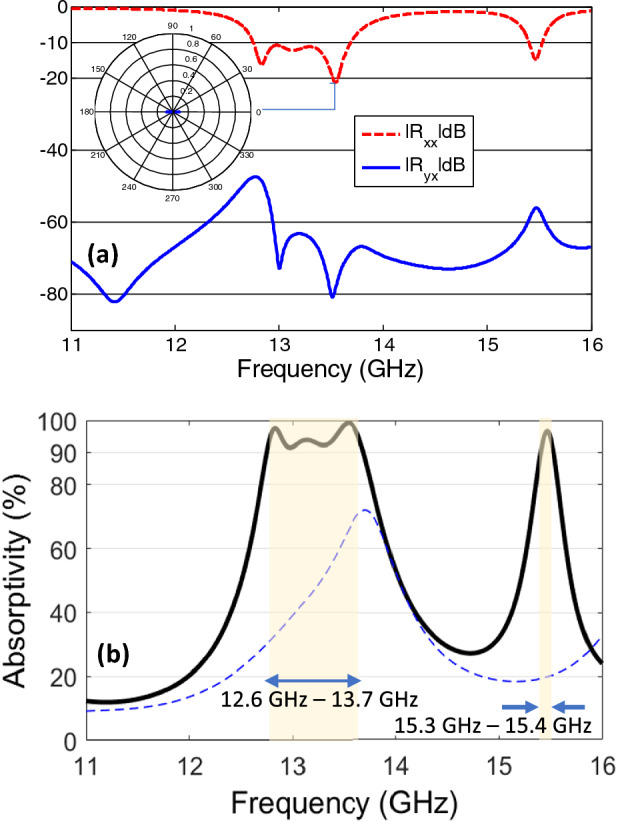
(**a**) Magnitude of reflection coefficients of co- and cross- polarized reflection for antireflecting metasurface. Inset shows the contour of time varying orientation of reflected electric field vector at resonance frequency. (**b**) Absorptivity spectrum of antireflecting metasurface absorber at normal incidence shown as solid black line. The near perfect absorption bandwidth region is highlighted. The absorptivity of co-directional split ring metasurface shown as dashed blue line is provided as reference.

It is clear that unidirectional metasurface supports polarization conversion from linear to cross polarized or circularly polarized reflected fields. As shown in schematic design illustration of Fig. 2 the rotation of the outer rings to form supercell with multidirectional splits can induce interesting resonant absorption phenomenon. The rotation allows induced currents to cancel each other and therefore minimize the radiated far field scattering (anti-reflection characteristics). Figure 4a shows that the magnitude of co- and cross- field components reflected field component reduce to -10 dB. The reason behind this resonant absorption is the destructive interference of induced currents on metasurface elements. Figure 4b shows that the absorptivity remains larger than $>90\%$ within frequency range of 12.6–13.7 GHz and 15.3–15.4 GHz. The bandwidth of near perfect level absorptivity greater than $>90\%$ is nearly 983 MHz (for 12.6–13.7 GHz resonant absorption band), whereas the Full Width at Half Maximum (FWHM) is found to be nearly 1.514 GHz. It is worth mentioning that the symmetry of the metasurface allows its response to be independent of incident polarization.

It is worth mentioning the comparison between resonant absorption levels for co-directional and rotated split ring metasurface. Since, the co-directional split ring metasurface works on the principle of resonance mechanism for generating chiral reflected fields. Therefore, it fails to achieve near perfect absorption and the absorptivity remains less than $70\%$ throughout the spectrum, see Fig. 4b. On the other hand, the rotated split-ring metasurface when arranged to induce anti-parallel surface currents leads to destructive interference for scattered fields. This allows the near perfect broadband absorption level at dual frequency bands. It is emphasized that the enhanced absorption level reached in this case is due to antireflection characteristics from the rotated split ring elements.

**Figure 5:**
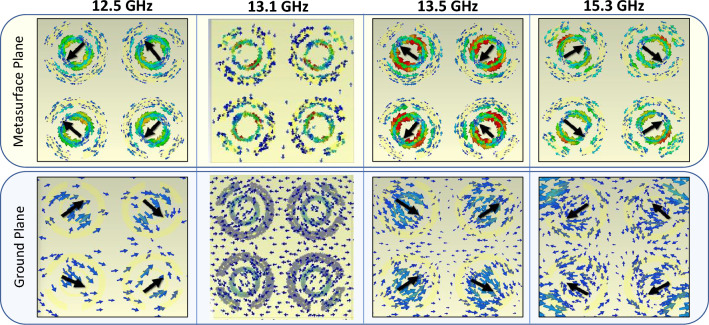
Surface current distribution for supercell at various frequencies 12.5 GHz, 13.5 GHz (absorption resonance) and 15.3 GHz (2nd narrowband absorption resonance).

Figure 5 shows the direction of surface currents on metasurface and ground planes at various resonant frequencies. The induced currents influence the overall scattering properties of the metasurface. The x-polarized incident fields coupled surface currents on metasurface and ground planes. As anticipated at resonance frequency the surface currents on metasurface and ground planes supports opposing currents (anti-parallel) on each cell that results in the cancellation of net dipole moment responsible for far field scattering.

**Figure 6:**
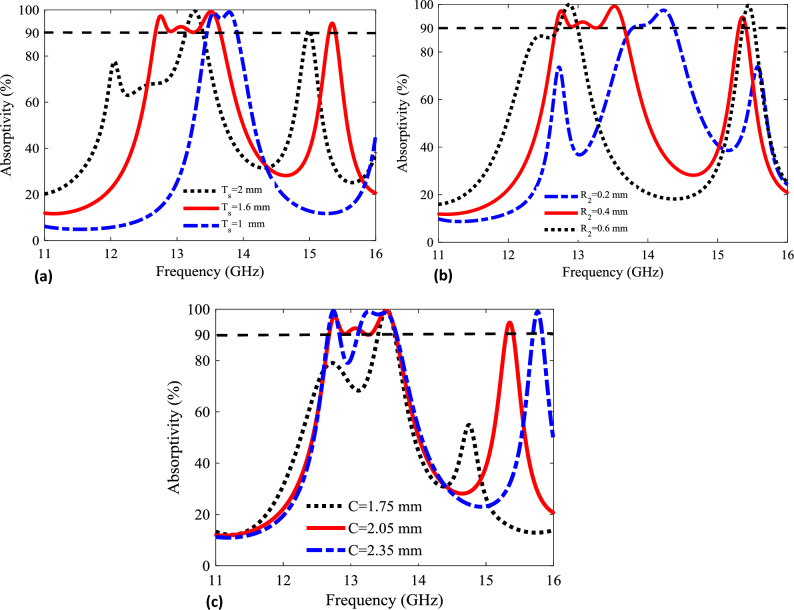
Absorptivity spectrum for different geometrical parameters including (**a**) thickness of substrate $T_s$, (**b**) radius of inner ring $R_2$ and (**c**) split ring gap size *C*. The schematic illustration of all such geometrical parameters in provided in Fig. 2.

It is interesting to determine the response of the metasurface due to variation in geometric parameters. From Fig. 6 it is clear that the resonant absorption response is sensitive to geometrical variation of the metasurface unit cell. Figure 6a provides the response of metasurface due to variation in thickness of substrate ($T_s$ = 1 mm, 1.6 mm, 2 mm) while keeping radius of inner ring ($R_2$) and split ring gap *C* as constants. Figure 6a shows that the thinner substrate thickness leads to blue-shift in resonance frequency along with variation in the bandwidth of absorption. The substrate thickness of 1.6 mm leads to optimized response with relatively broadband resonant absorption. Hence, there is inverse relation between substrate thickness and resonant frequency. Therefore, the substrate thickness has to be lower for the patch to be resonating at higher frequency. Another important geometric parameter is the arm width given as radius ($R_2$). Figure 6b shows effect of resonant absorption due to variation in the radius ($R_2$ = 0.2 mm, 0.4 mm, 0.6 mm) of inner ring. The smaller ring radius leads to blue-shift in the resonant frequency along with variation in resonance bandwidth. The shift in the resonant frequency is observed for smaller $R_2$ but at a cost of reduced bandwidth. The optimized value for arm width of inner ring is $R_2$ = 0.4 mm. Finally, the effect of variation in gap size of split ring (*C* = 1.75 mm, 2.05 mm, 2.35 mm) is analyzed in Fig. 6c. It is clear that reduction in gap size effects the resonant coupling and a red-shift is observed for narrowband resonance for smaller split ring gap size (*C*). The optimized value for split ring gap is *C* = 2.05 mm.

**Figure 7:**
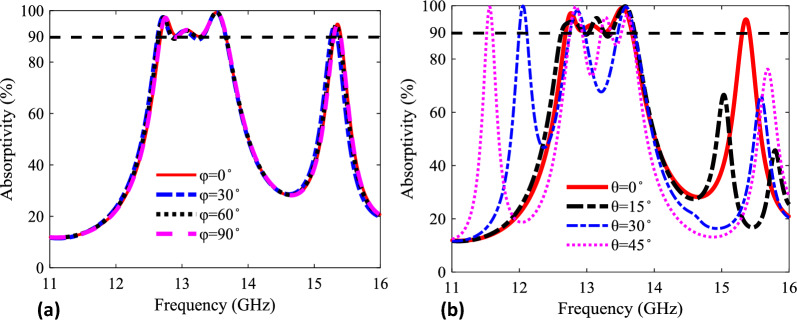
Absorptivity spectrum for different incident direction along (**a**) azimuthal angle ($\phi$) ranging from 0° to 90° and (**b**) polar angle ($\theta$) ranging from 0° to 45°.

The absorptivity spectrum can also be determined for oblique incidence condition. Figure 7 shows the variation in absorptivity spectrum due to oblique incidence. The variation in oblique incidence is provided for two different conditions i.e., azimuthal angle considering electric field is in the plane or polar angle considering magnetic field is in the plane of metasurface. Figure 7a shows that the metasurface response for in-plane electric field configuration. The absorptivity remains stable for azimuthal angle supporting a wide polarization angle insensitivity for varying angle between 0° to 90°. Furthermore, it is emphasized that the structural symmetry allows identical response for x- and y-polarized incident waves that allows similar response of metasurface from arbitrary incident direction. On the other hand, the Fig. 7b shows the response of metasurface for in-plane magnetic field configuration. The absorptivity remains stable for polar angle with appearance of additional resonance bands at higher oblique angles.

**Figure 8:**
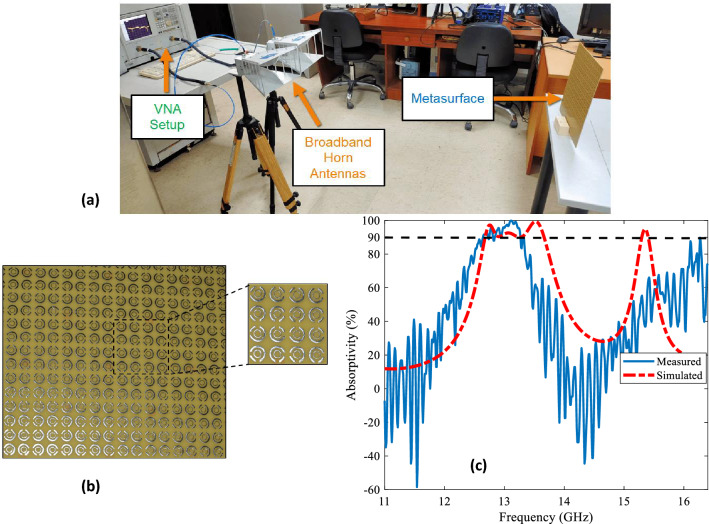
(**a**) Experimental setup for the measurement of co-polarized reflection component $R_{xx}$. For cross polarized reflection component, the receiving horn antenna is rotated by 90°. (**b**) The fabricated prototype of the proposed metasurface absorber. (**c**) A comparison between measured and simulated absorptivity spectrum for the anti-reflecting metasurface absorber.

The measurements of metasurface absorber have been carried out in the RIMMS laboratory. Figure 8a shows the setup for the measurement of metasurface that is comprised of two broadband horn antennas connected to a vector network analyzer (VNA). The VNA acts as a signal source and excites the horn antennas for transmitting and receiving electromagnetic waves. Agilent E8362B vector network analyzer was used for the measurements. The proposed metasurface is fabricated using standard printing circuit board technique on 1.6 mm thick FR-4 sheet. The physical size of the sheet is 30 cm × 30 cm while its electrical size is 27$\lambda$ $\times$ 27$\lambda$ with $\lambda$ = 1.1 cm at the centered operating frequency of 13 GHz of the operating band 12.687–13.669 GHz. The fabricated design is shown below in Fig. 8b. As the measurements are performed in the open-lab environment, therefore the measurements were also performed for the lab environment without metasurface to mitigate the surrounding effects. A comparison between simulated and measured reflection coefficients and the resulting absorptivity is provided in Fig. 8c.

It can be observed that the results have good agreement except some jittering effect in the measured results. considering the finite size of metasurface with horn antennas having larger aperture likely causes a deviation in the response when compared to simulations that assumes periodic arrangement of unitcells with incidence under planewave conditions. The measured S-parameters have some noisy peaks and different types of filtering algorithms can be used for the statistical treatment of the measured data to remove the noise from the measured data. The misalignment between the metasurface and horn antennas can lead to slight deviations in the measured results due to angular instability.

## Conclusion

In this paper, a single layer and polarization-independent metasurface absorber is proposed without using lumped elements or resistive sheets. The metasurface absober design is based on distinct supercell approach that works through the destructive interference of antiparallel electromagnetic scattering emerging from chiral cell elements. A single substrate layer of FR-4 sandwiched between the top and bottom metal layers was used to achieve broadband. A single layer design without lumped elements results in low cost and easy fabrication process of the design. The proposed absorber showed broadband of 983 MHz without using lumped elements, resistive sheets or multilayer design. The absorption bandwidth is greater than 90$\%$ for dual bands. The Full Width at Half Maximum (FWHM) is reported to be 1.514 GHz. A prototype of the proposed design is fabricated and tested for absorption and polarization stability. The design shows good agreement between measurement and simulation results. This design has various applications in EMC/EMI, RCS reduction, satellite and stealth technology.

## Data Availability

All data required to evaluate the findings of this work is available in the presented paper. Additional data related to this work may be requested from the corresponding author.
