# Author Correction: Anti-reflecting metasurface for broadband polarization independent absorption at Ku band frequencies

**DOI:** 10.1038/s41598-023-35750-z

**Published:** 2023-06-01

**Authors:** Muhammad Amin, Aliza Fida, Aamir Rashid, Omar Siddiqui, Farooq A. Tahir

**Affiliations:** 1 College of Engineering, Taibah University, Madinah, 41411 Saudi Arabia; 2 Department of Electronics Engineering, University of Engineering and Technology, Taxila, Pakistan; 3 School of Electrical Engineering and Computer Science, National University of Sciences and Technology (NUST), Islamabad, Pakistan

Correction to: *Scientific Reports* 10.1038/s41598-022-24691-8, published online 22 November 2022

Aliza Fida was omitted from the author list in the original version of this Article.

The Author Contributions section now reads:

“M.A., O.S. and F.A.T. conceived the idea of anti-reflecting metasurface for absorption. M.A. and O.S. analyze the results and wrote the manuscript. A.F, A.R. and F.A.T. performed simulations, fabrication and measurements. F.A.T. also contributed to the manuscript writeup and supervised the whole research.”

The original Article has been corrected.

